# Investigating Friendship Difficulties in the Pathway from ADHD to Depressive Symptoms. Can Parent–Child Relationships Compensate?

**DOI:** 10.1007/s10802-021-00798-w

**Published:** 2021-03-02

**Authors:** Victoria Powell, Lucy Riglin, Terry Ng-Knight, Norah Frederickson, Katherine Woolf, Chris McManus, Stephan Collishaw, Katherine Shelton, Anita Thapar, Frances Rice

**Affiliations:** 1 Cardiff University, Cardiff, UK; 2 University of Surrey, Guildford, UK; 3 University College London, London, UK

**Keywords:** ADHD, Depression, Friendship, Peer, Parent–child relationship, Transition

## Abstract

Attention deficit/hyperactivity disorder (ADHD) is associated with friendship difficulties. This may partly account for the increasingly recognised association between ADHD and subsequent depression. Little is known about the types of friendship difficulties that could contribute to the association between ADHD and depressive symptoms and whether other relationships, such as parent–child relationships, can mitigate against potential adverse effects of friendship difficulties. In a representative UK school sample (*n* = 1712), three main features of friendship (presence of friends, friendship quality and characteristics of the individual’s classroom friendship group) were assessed in a longitudinal study with two assessment waves (W1, W2) during the first year of secondary school (children aged 11-12 years). These friendship features (W1) were investigated as potential mediators of the prospective association between teacher-rated ADHD symptoms (W1) and self-rated depressive symptoms (W2) seven months later. Parent–child relationship quality (W1) was tested as a moderator of any indirect effects of ADHD on depression via friendship. ADHD symptoms were inversely associated with friendship presence, friendship quality and positive characteristics of classroom friendship groups. Depressive symptoms were inversely associated with presence and quality of friendships. Friendship quality had indirect effects in the association between ADHD and subsequent depressive symptoms. There was some evidence of moderated mediation, whereby indirect effects via friendship quality attenuated slightly as children reported warmer parent–child relationships. This highlights the importance of considering the quality of friendships and parent–child relationships in children with ADHD symptoms. Fostering good quality relationships may help disrupt the link between ADHD symptomology and subsequent depression risk.

**Supplementary Information:**

The online version contains supplementary material available at 10.1007/s10802-021-00798-w.

## Introduction

Attention Deficit/Hyperactivity Disorder (ADHD) is a DSM-5 (American Psychiatric Association, 2013) defined neurodevelopmental disorder that is associated with a range of long term adverse outcomes, including employment and education difficulties, interpersonal problems, substance abuse, suicide and depression (Harpin, 2005; Ljung et al., 2014; Meinzer et al., 2014). There is strong evidence that ADHD precedes depression in a way that is consistent with a potentially causal relationship (Riglin et al., 2020), but the mechanisms that might explain the relationship between ADHD and later depression are unclear. ADHD impacts on many aspects of a young person’s life, including functioning in social, school and home life (Harpin, 2005), that might in turn increase the risk of subsequent depression. For instance, ADHD is associated with increased difficulties in friendships (Mikami, 2010). Studies comparing children with an ADHD diagnosis to typically developing controls show that children with ADHD are likely to have fewer stable friendships (Blachman & Hinshaw, 2002; Marton et al., 2015) and may be more likely to be friends with individuals reported as having a learning or behaviour problem (Marton et al., 2015). Friendship difficulties such as poor quality friendships may be risk factors for subsequent depression (Goodyer et al., 1989).

One potential explanation of the prospective association of ADHD and depression is that social stressors that commonly accompany ADHD, such as friendship difficulties, lead to an increased risk of depression (Capaldi, 1992), potentially by creating feelings of failure or lowered self-esteem (Cole, 1990; Patterson & Stoolmiller, 1991). Interpersonal stress is an important precipitator of depression, particularly during adolescence (Flynn & Rudolph, 2011) – a period when individuals spend increasing amounts of time with friends (Larson & Richards, 1991). Studies have shown that bullying (Roy et al., 2015), social stress (Humphreys et al., 2013) and peer-relationship difficulties (Powell et al., 2020) contribute to the prospective relationship of ADHD and depressive symptoms in young people. However, a detailed investigation of friendship features including presence of friends, friendship quality and characteristics of the friendship group (Bukowski et al., 1996) and how these are associated with ADHD and impact on later depression is lacking (Mikami, 2010; Mrug et al., 2012). Important features of friendship include whether an individual has friends, the quality of these friendships including the level of companionship and closeness, as well as the characteristics of the people that an individual is friends with (Bukowski et al., 1996). It is possible that some of these features of friendship might be more important than others in the association of ADHD and depression. For instance, a previous study conducted in secondary school children found that while retaining your best friend over time was not associated with emotional outcomes, retaining poor quality friendships over time with your top three friends was associated with subsequent emotional problems (Ng-Knight et al., 2019). The potential role of friendships in the prospective association between ADHD and depression may also differ by sex. For example, adolescent females have been found to value aspects of friendship such as companionship more highly than males, while adolescent males have been found to value the status of their peers more highly (Hall, 2010). Identification of factors that might explain the relationship between ADHD and depression, as well as factors that moderate risk, could help pinpoint new ways of supporting young people with ADHD.

School is a key context where children make friends (Ng-Knight et al., 2019). Classroom expectations including sustained concentration on tasks, following rules and self-regulation of emotions and behaviour may often be a poor ‘fit’ for those with ADHD, which can exacerbate poor social outcomes for this group (Richardson et al., 2015). The transition from primary to secondary school is a period of change (Chung et al., 1998) when good friendships can protect against poor mental health (Ng-Knight et al., 2019), but also when there is a natural disruption to established friendships and a need to establish new ones.

In addition to friendships, it is important to consider children’s other sources of social support including parent–child relationships, which can mitigate against poor mental health outcomes in the presence of adversity (Collishaw et al., 2016). Some research suggests a compensatory model, whereby children with low levels of warmth from peers showed better adjustment outcomes in the presence of high parental warmth compared to low parental warmth (Stocker, 1994). Warmer relationships with parents are also associated with more satisfactory friendships in young people (Deković & Meeus, 1997), and the influence of parental behaviour on the child’s friendships may be particularly important in children with ADHD (Mikami et al., 2010). Thus, it is possible that any mediating effects of friendship in the association of ADHD and depression could be moderated by the quality of the parent–child relationship. However, parent–child relationships are often not considered in studies of ADHD and friendship (Mikami, 2010) and this is especially the case for the father-child relationship (Cabrera et al., 2018). Investigating the influence of relationships with both mother and father is important, as they may exert differential influences on children’s friendships (Flynn et al., 2018; Updegraff et al., 2001). For example, a previous study found that mother supportive behaviour and hostile behaviour, and father problem-solving behaviour and hostile behaviour, influenced their adolescent children’s interaction styles with peers (Flynn et al., 2018).

The aim of this study was to investigate which aspects of friendship (presence of friends, friendship quality and characteristics of friends) are important in increasing vulnerability to depressive symptoms in those with elevated ADHD symptoms. A secondary aim of this study was to investigate whether any indirect effects via elements of friendship were moderated by mother–child or father-child relationship quality. A follow-up exploratory research question was whether indirect effects differed according to sex. The study included children in the first year of secondary school followed over a seven-month timespan.

## Methods

### Sample

The School Transition Adjustment Research Study (STARS; Ng-Knight et al., 2016) includes pupils recruited from nine secondary schools in Greater London selected to be representative of schools in the local region in terms of socioeconomic disadvantage, exam pass rates and proportions of pupils from minority ethnic backgrounds. Ethical approval was granted by the University College London Research Ethics Committee. At both assessment waves, informed assent was obtained at school for the participating children and parents were given the opportunity to opt-out. Baseline data were collected during the second half of the first term of year 7 (children aged 11-12 years; pupil and teacher reports) and follow-up data seven months later in the summer term of year 7 (pupil reports) via questionnaires completed in classroom settings. At baseline, 1712 children participated (53.5% male; response rate = 87.0%). For 80.1% of these children, teachers returned questionnaires reporting on the child’s mental health (*n* = 1372). For 1020 of these 1372 children (74.3%), teachers completed the section rating the children’s baseline ADHD symptoms. Of the 1020 children, self-rated depressive symptom data at follow-up were available for 901. Of these, there were 752 (50.3% male) for whom data were additionally available on friendship and sociodemographic variables. To address bias that might be caused by missingness, predictors of missingness were investigated (Supplement 1) and missing data were imputed for covariates and outcome using Multiple Imputation by Chained Equations (MICE; White et al., 2011) with 100 imputations, which formed the analysis sample (*n* = 1712; Supplement 2).

### Measures

#### ADHD Symptoms

ADHD symptoms were measured at baseline with the teacher-rated hyperactivity-inattention subscale of the Strengths and Difficulties Questionnaire (SDQ; Goodman, 1997). Five items were rated “Not True” (0), “Somewhat True” (1) or “Certainly True” (2) (total score range = 0–10; Cronbach alpha = 0.82). The teacher-rated hyperactivity-inattention subscale is a useful and valid screening tool for ADHD symptoms (Goodman, 1997) with good sensitivity and specificity for detecting DSM-IV (American Psychiatric Association, 1994) ADHD in school-aged children (Goodman et al., 2000; Ullebø et al., 2011). In UK secondary schools, children are typically taught by different teachers for different subjects. Children usually attend a daily registration group led by the same teacher throughout the school year (the form tutor). Form tutors completed the teacher questionnaire, including the SDQ.

#### Depressive Symptoms

Depressive symptoms were assessed at follow-up with the self-rated Short Moods and Feelings Questionnaire (SMFQ; Angold et al., 1995). Thirteen items were rated “Not true” (0), “Somewhat true” (1), or “True” (2) (total score range = 0–26; Cronbach alpha = 0.91). The SMFQ is a well-validated, reliable measure of depression in adolescents with high sensitivity and specificity for detecting major depressive disorder (MDD) (Thapar & McGuffin, 1998).

#### Friendship Presence

At baseline and follow-up, children were asked to name up to three friends in order of preference as their top 3 friends. This method was used to indicate the presence of close friends, as used previously (e.g., Fowler et al., 2007; Ng-Knight et al., 2019). The following variables were derived: number of friends at baseline (range = 0–3), stability of best friend (from the start to the end of the school year; 0 = no, 1 = yes) and stability of ‘top three friends’ (stability of any of the three top friends; 0 = no, 1 = yes). This method of deriving friendship stability has also been used previously (Ng-Knight et al., 2019).

#### Friendship Quality

For their best friend, children completed a 15-item version of the Friendships Qualities Scale, a reliable measure of five different aspects of friendship quality, at baseline (FQS; Bukowski et al., 1994). The FQS consists of five subscales: companionship, conflict, closeness, help and security. Items were rated on a 5-point Likert scale from “Not at all” (0) to “Very much” (4). Positive scales (companionship, closeness, help and security) were summed and then negative scales (conflict) subtracted to generate a maximum total quality score of 48. The Cronbach alpha suggested that this score is reliable (0.85), as did the omega value (0.85), which does not assume one-dimensionality or equal contribution of each item to the total score (Deng & Chan, 2017). In addition, friendship quality across the top three friends was measured at baseline by a single rating for each of these friends on a 5-point smiley face rating scale from 0 (least satisfied) to 4 (most satisfied). Scores for the top three friends were summed to give an overall score (maximum = 12).

#### Classroom Friendship Group Characteristics

Sociometric methods were used to identify classroom friendship groups at baseline. In classroom groups, children were asked to draw on paper a diagram describing “which children in your class hang around together” by circling groups of pupils who are friends (example shown in Supplement 3). Social Cognitive Mapping software (SCM; Cairns & Cairns, 1994; Hamm et al., 2011) was used to identify classroom friendship groups – a reliable, valid tool for this purpose (Cairns et al., 1988, 1995). To describe the characteristics of the classroom friendship group, this was linked to information on the behavioural and emotional characteristics of children in that group: (i) the self-rated SDQ total difficulties score (Goodman, 1997), and (ii) cooperative and disruptive behaviour rated by peers who indicated from a list of the children in their class who they felt matched these descriptions (example shown in Supplement 4; the Guess Who peer-nomination method; Coie & Dodge, 1988; Parkhurst & Asher, 1992). Classroom friendship group characteristics were derived for each child by averaging the scores of the other pupils in their friendship group (the score of the individual was not included to avoid group scores being biased by the individual’s own score). The mean number of individuals in a classroom friendship group was 5 (maximum: 15). A small number (5.3%) of children were social isolates (i.e., did not have a classroom friendship group) so their scores could not be calculated.

#### Parent–Child Relationship Quality

At baseline, children who reported being in contact with their mother and/or father (or equivalents, e.g., stepparent, carer) in the last month completed a measure assessing their perception of each parent’s warmth and hostility towards them using the Iowa family interaction rating scale (Melby & Conger, 2001). The warmth scale consisted of six items (example: “Acts loving and affectionate toward you”) rated on a 6-point Likert scale from “Never” (0) to “Always” (6) (score range = 0–36; Cronbach’s alpha for mother score = 0.90; Cronbach’s alpha for father score = 0.92). The hostility scale consisted of 4 items (example: “Criticises you or your ideas”) rated in the same way (score range = 0–24; Cronbach’s alpha for mother score = 0.79; Cronbach’s alpha for father score = 0.82).

#### Confounders

Analyses were adjusted for sex and socioeconomic and ethnic factors associated with depression (Gilman et al., 2002; Williams et al., 2015). These were socioeconomic disadvantage (free school meals status), Black Minority Ethnic status and English as a first language (data collected from school records).

### Data Analysis

Analysis was conducted in STATA (version 13). All analyses presented are adjusted for confounders by including these variables in the models as covariates.

#### Associations of ADHD, Features of Friendship and Depression

ADHD symptoms were standardised meaning a point increase in SDQ hyperactivity score was equivalent to a standard deviation unit increase. Linear regression was used to test the association between ADHD symptoms and depressive symptoms 7 months later. Separate linear and logistic regressions were used as appropriate to test associations between ADHD symptoms and indicators of three friendship elements (presence/stability, quality, and characteristics of friends), and to test associations of these friendship indicators (standardised if continuous) with depressive symptoms. Regressions included the school class as a random effect to account for potential hierarchical data structure or clustering.

#### Indirect Effects via Friendship in the ADHD-Depression Association 

To test whether the different features of friendship contributed to the association of ADHD and depressive symptoms, indirect effects were tested separately using the [sureg] STATA command. Sureg conducts Seemingly Unrelated Regressions (Zellner, 1962), from which indirect (mediated) and conditional indirect (moderated mediation) effects can be derived in original and imputed data (UCLA Statistical Consulting Group n.d.). Any friendship variables found to have significant indirect effects were tested simultaneously in a multiple mediator model. As indirect effects via friendship may differ according to sex (Hall, 2010), a sensitivity analysis testing moderated mediation by the child’s sex was conducted using [sureg].

#### Moderation of Indirect Effects by Parent–Child Relationships

Moderated mediation was tested using [sureg] to investigate whether any indirect effects via friendship varied according to the warmth or hostility of parent–child relationships. The model used tested combined moderation of the pathway from ADHD symptoms to friendship and the pathway from friendship to depression symptoms (Preacher et al., 2007). Indirect effects at the mean level of the moderator and 1 standard deviation above and below this were calculated and plotted. For each moderator, significance tests (*Z*-tests) of the difference between the observed indirect effect at the mean level of the moderator compared to the mean level of the moderator -1SD (for hostility) or +1SD (for warmth) were conducted. To investigate which path(s) in the indirect effects were being moderated, we additionally tested models with an interaction effect on the path between ADHD symptoms and friendship or the path between friendship and depression symptoms only.

## Results

Descriptive statistics are shown in Table 1.

**Table 1:** Descriptive Statistics

Variable	Overall mean (SE) / proportion (%)	Female mean (SE) / proportion (%)	Male mean (SE) / proportion (%)
ADHD	1.97 (0.07)	1.33 (0.08)	2.53 (0.11)
Depression	3.36 (0.12)	3.85 (0.19)	2.94 (0.16)
Presence of friends	2.90 (0.01)	2.93 (0.01)	2.87 (0.02)
Stability: best friend	39.6%	42.1%	37.5%
Stability: top three friends	62.3%	65.2%	59.8%
Quality: best friend	34.81 (0.21)	37.14 (0.28)	32.78 (0.30)
Quality: top three friends	10.84 (0.04)	10.81 (0.05)	10.86 (0.05)
Classroom friendship group: total difficulties	7.99 (0.20)	7.18 (0.22)	8.70 (0.24)
Classroom friendship group: cooperativeness	0.51 (0.01)	0.54 (0.01)	0.47 (0.01)
Classroom friendship group: disruptiveness	0.12 (0.005)	0.07 (0.005)	0.17 (0.01)
Number of people in classroom friendship group	4.98 (0.07)	4.59 (0.08)	5.33 (0.10)

*n* = 1712 (53.5% male), *ADHD* attention deficit/hyperactivity disorder, *SE* standard error

### Associations of ADHD, Features of Friendship and Depression

ADHD symptoms at baseline were associated with naming fewer friends and lower friendship quality at baseline, both of which were associated with increased depressive symptoms at follow-up. ADHD symptoms were also associated with being part of a classroom friendship group that had higher total difficulties and was rated as less cooperative and more disruptive at baseline. Stability of the best friendship from baseline to follow-up was inversely associated with depressive symptoms (Table 2). ADHD symptoms were associated with depressive symptoms 7 months later (*b* = 0.48 (95% CI 0.17, 0.79) *p* = 0.002).

**Table 2:** Associations of ADHD Symptoms, Friendship Variables and Depressive Symptoms

Friendship variable	ADHD symptoms association with variable (*b* (95% CI) *p*)	Variable association with depressive symptoms (*b* (95% CI) *p*)	Indirect effect via variable between ADHD and depressive symptoms (*b* (95% CI) *p*)
Presence of friends	-0.05 (-0.08, -0.02) 0.002	-0.65 (-1.24, -0.07) 0.029	0.03 (-0.004, 0.06) 0.095
Stability: best friend	OR = 0.92 (0.80, 1.06) 0.267	-0.57 (-1.07, -0.06) 0.027	0.01 (-0.01, 0.03) 0.262
Stability: top three friends	OR = 0.96 (0.82, 1.12) 0.568	-0.32 (-0.84, 0.21) 0.235	0.004 (-0.01, 0.02) 0.530
Quality: best friend	-0.75 (-1.29, -0.20) 0.008	-0.72 (-0.97, -0.47) < 0.001	0.06 (0.01, 0.11) 0.011
Quality: top three friends	-0.15 (-0.25, -0.05) 0.003	-0.69 (-0.94, -0.44) < 0.001	0.07 (0.02, 0.11) 0.008
Classroom friendship group: total difficulties	0.94 (0.61, 1.27) < 0.001	0.17 (-0.10, 0.43) 0.211	0.02 (-0.05, 0.08) 0.633
Classroom friendship group: cooperativeness	-0.03 (-0.04, -0.02) < 0.001	-0.03 (-0.29, 0.23) 0.798	-0.01 (-0.06, 0.04) 0.663
Classroom friendship group: disruptiveness	0.03 (0.02, 0.04) < 0.001	0.15 (-0.12, 0.42) 0.262	-0.01 (-0.05, 0.08) 0.743

*n* = 1712, *ADHD* attention deficit/hyperactivity disorder, *b* unstandardized beta, *CI* confidence interval, *OR* odds ratio

### Indirect Effects via Friendship in the ADHD-Depression Association

ADHD and depressive symptoms were associated directly and indirectly via friendship quality, both for best friend and top three friends, when tested separately (Table 2). They showed independent effects when tested simultaneously in a multiple mediator model (Table 3), each accounting for approximately 9% of the total effect of ADHD symptoms on subsequent depressive symptoms. Sensitivity analyses suggested that the best friendship quality sub-scales of higher friendship conflict and lower friendship security drove this effect (Supplement 5).

**Table 3:** Indirect Effects via Friendship Quality and Satisfaction in the Association of ADHD and Depressive Symptoms in a Multiple Mediator Model

Effect	*b* (95% CI)	*p*	Percentage of total effect mediated
Indirect Effect Via Best Friendship Quality	0.04 (0.01, 0.08)	0.024	9.05%
Indirect Effect Via Top Three Friendships Quality	0.05 (0.006, 0.09)	0.024	9.48%
Total Indirect Effect	0.09 (0.03, 0.15)	0.002	18.53%
Direct Effect	0.39 (0.09, 0.70)	0.011	-
Total Effect	0.48 (0.18, 0.79)	0.002	-

*n* = 1712, *ADHD* attention deficit/hyperactivity disorder, *b* unstandardized beta, *CI* confidence interval

The indirect effect of ADHD symptoms on depressive symptoms via friendship quality was larger for females than males (Supplement 6).

### Moderation of Indirect Effects by Parent–Child Relationships

**Fig. 1:**
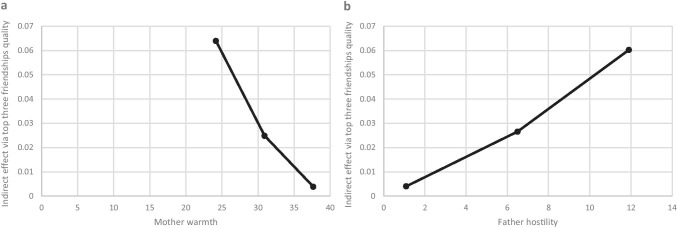
Plots of Moderated Mediation. Moderated mediation analysis suggested that when mother warmth increased, the indirect effect (beta) via quality of friendship with top three friends between ADHD and depressive symptoms attenuated (**a**). When father hostility decreased, the indirect effect via quality of friendship with top three friends also attenuated slightly (**b**), though this did not meet conventional thresholds for statistical significance. The model tested included interaction effects on the path between ADHD symptoms and friendship quality and on the path between friendship quality and depressive symptoms. Indirect effect betas at the mean of the moderator and the mean ± 1 standard deviation are plotted (*n* = 1712)

**Fig. 2:**
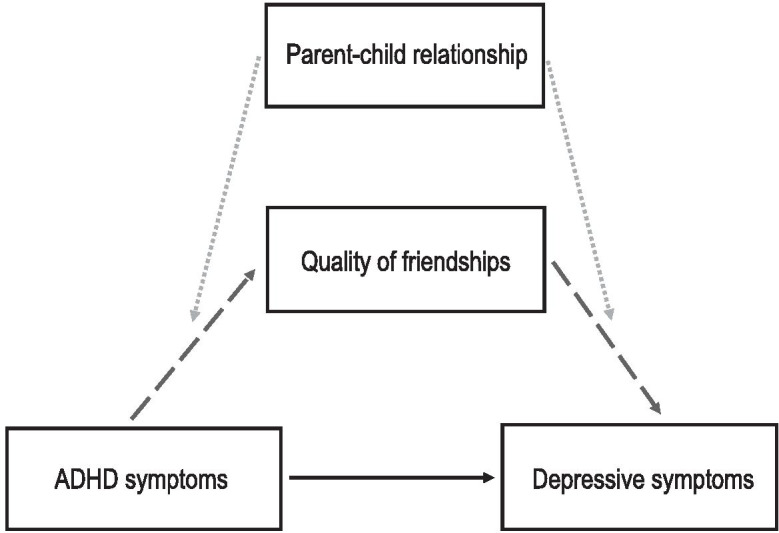
Moderated Mediation Model. Results supported a model whereby friendship quality accounted for part of the association between ADHD symptoms and depressive symptoms (black arrow) via mediated pathways (dashed dark grey arrows). There was some evidence to suggest that the indirect effect via top three friendships quality might be moderated by parent–child relationships (dotted light grey arrows), whereby warmer, less hostile relationships with mothers and fathers slightly decreased the size of the indirect effect

Most (98.0%) children reported that they had been in contact with their mother in the last month and 92.5% with their father. Indirect effects via top three friendships quality attenuated (*p* = 0.040) as mother–child relationship warmth increased (Fig. 1; Supplements 7 & 8). Results were suggestive of this moderating effect by mother warmth acting on both paths in the indirect effect via friendship quality between ADHD and depressive symptoms (Fig. 2; Supplement 8). Indirect effects via top three friendships quality also attenuated slightly as father-child hostility decreased, though this observation did not reach the conventional threshold for statistical significance (*p* = 0.084) (Fig. 1; Supplement 7).

All results were very similar before and after imputation (Supplement 9).

## Discussion

To our knowledge, this is the first detailed investigation of how different aspects of friendships are involved in the prospective association of ADHD and depressive symptoms. We also investigated whether parent–child relationship quality moderated any indirect effects via friendship in the prospective association of ADHD and depressive symptoms. In a representative longitudinal study of children, ADHD symptoms were associated with children having fewer friends and lower quality friendships, which were both associated with depressive symptoms. ADHD symptoms were also associated with having a classroom friendship group that had more total difficulties, was more disruptive and less cooperative. Retaining best friendships across the study period was inversely associated with depression symptoms. These findings build upon case–control studies that found an association between ADHD and having fewer friends, more friendship conflict and aggression (Blachman & Hinshaw, 2002) and an increased likelihood of being friends with a child with a learning or behaviour problem (Marton et al., 2015), and an association between poor friendship quality and depression (Goodyer et al., 1989). Despite these varied associations, only friendship quality was identified as a pathway through which ADHD symptoms were associated with subsequent depressive symptoms. The indirect effect via top three friendships quality varied slightly in magnitude according to the warmth and hostility of parent–child relationships, suggesting that positive parent–child relationships might mitigate some of the adverse effects in the indirect pathway of ADHD to depressive symptoms via poor quality friendships.

The findings on friendship quality align with theories of social difficulty explaining some of the link between early psychopathology and subsequent depression (Capaldi, 1992). Our findings suggest this is also a pathway that links ADHD to depression symptoms. Friendship quality as a form of perceived social support might reduce depression risk in those with elevated ADHD symptoms via increasing a sense of connectedness and self-esteem, or by buffering against life stresses (Rueger et al., 2016). Those with ADHD are more likely to experience adversity in various areas of life (Harpin, 2005) and good social support can mitigate against depressive outcomes in those who experience adversity (Collishaw et al., 2016; Lee et al., 2019). Sensitivity analyses showed that conflict and security with friends (ability to disclose problems to friend and reconcile after disagreement) appeared to be the specific elements of friendship quality that were important in the pathway from ADHD to depressive symptoms. Elevated conflict with friends has been reported for children with ADHD (Blachman & Hinshaw, 2002) and poor-quality friendships may also be risk factors for depressive outcomes in school aged children (Goodyer et al., 1989).

Larger indirect effects via friendship quality were observed in females than males. This aligns with previous evidence of females valuing aspects of friendship quality such as companionship and intimacy more highly than males (Hall, 2010). Interpersonal stress may be more prevalent and predictive of depression in adolescent females than males (Shih et al., 2006), particularly in females with ADHD, who may experience more peer-relationship difficulties than males with ADHD (Elkins et al., 2011). However, it seems likely that the indirect effects observed are important to consider for both sexes.

There was some evidence of moderated mediation suggesting that indirect effects of top three friendships quality on the link between ADHD and depressive symptoms decreased slightly as self-reports of mother–child relationship warmth increased and father-child relationship hostility decreased, though the evidence for father hostility did not reach the conventional significance threshold. This draws attention to the need to consider the child’s social support across different contexts such as the parent–child relationship. Findings were suggestive of mother warmth moderating both the ADHD symptoms to friendship quality path and the friendship quality to depression symptoms path of the indirect effect. This finding is consistent with that of a previous study that found an association between parental behaviour and child peer-relationships in children with ADHD (Mikami et al., 2010) in addition to studies that have found parent–child relationships may be able to compensate for a lack of friends (Stocker, 1994) and mitigate against poor mental health in the presence of adversity (Brennan et al., 2003; Collishaw et al., 2007, 2016; Lewandowski et al., 2014). However, these moderated mediation findings should be interpreted with caution given that results were not consistent across mother and father or between warmth and hostility. Nevertheless, this may be explained in part by previous findings that suggest mothers and fathers may have differential effects on the friendships of their adolescent children (Flynn et al., 2018; Updegraff et al., 2001). For instance, one study found that while mother supportive behaviour and hostile behaviour influenced their children’s interactional style with peers, for fathers, it was their problem-solving behaviour and hostile behaviour that appeared to be important (Flynn et al., 2018). Moderated mediation results in the current study were also inconsistent across the two friendship quality measures used. While some evidence of moderation by mother warmth was observed for the indirect effect via friendship quality with the top three friends, strong evidence of moderation by parent–child relationship quality was not found for the indirect effect via friendship quality with the best friend. Measurement differences in the two friendship variables used in the present study may have affected the findings, as a previous study found evidence of an interaction of friendship stability with a variable measuring quality of top three friendships, which was not found for the quality of the best friendship only (Ng-Knight et al., 2019).

Limitations include that we relied on teacher reports of child ADHD symptoms which provide a reliable measure of ADHD symptoms in school, but most clinical research and practitioners rely primarily on parental reports of symptoms. In addition, while the SDQ hyperactivity-inattention subscale completed by teachers is a valid and useful screening tool for ADHD (Goodman, 1997), it is not a diagnostic measure and we were not able to investigate whether ADHD inattentive or hyperactive-impulsive subtypes were differentially associated with friendship or depression in this study. The inattentive subtype has been found to be the most common subtype in population samples, particularly during adolescence (Willcutt, 2012). Associations between ADHD and emotional and peer problems may vary according to subtype (Graetz et al., 2001), and thus subtype differences are of interest for future research. Despite the longitudinal design used, there is a possibility that reverse causation contributed to observed associations. However, evidence suggests that ADHD precedes depression in a potentially causal relationship (Riglin et al., 2020) and that the prospective relationship exists over long periods of time and when adjusting for prior emotional disorder symptomatology (Powell et al., 2020). Moreover, a sensitivity check found that ADHD symptoms were associated with subsequent depressive symptoms when adjusting for baseline self-reported SDQ emotional problems (Goodman, 1997; Supplement 9). Although we tested indirect effects, this is not mediation analysis per se, due to exposure and mediator being measured contemporaneously (Selig & Preacher, 2009). This study had missing data, a common problem in longitudinal data (Spratt et al., 2010). However, tests were adjusted for confounders that predicted missingness, helping to address potential bias arising from missing data (Groenwold et al., 2012). Additionally, we conducted Multiple Imputation and results remained very similar in imputed data, suggesting bias caused by missingness was minimal (Spratt et al., 2010). Those with ADHD may under-report their depressive symptoms and over-report their social competence, while those with depression might under-rate their social ability, which could attenuate associations (Fraser et al., 2018; Ohan & Johnston, 2011; Whitton et al., 2008). In addition, we were unable to investigate whether the friendships reported by the children were reciprocated in the current study, due to reported best friends not necessarily attending the same school. However, children were asked to report only their top three friendships – an approach that has been used previously (Fowler et al., 2007; Ng-Knight et al., 2019). Children were asked to report their best three friends rather than to report a total number of friends to capture the children’s close friendships, thereby helping to avoid some of the positive illusory bias that may affect reporting on friendships in children with ADHD symptoms. While measures of how many friends a child has in total may capture how popular or liked that individual is on a group level (e.g., in their class or school), measures that capture children’s close friendships may be more predictive of later adjustment and depression (Narr et al., 2019; Schneider et al., 1994). In addition, while self-rated measures of friendship presence and quality were used, peer-rated classroom data was also used to measure the characteristics of the friendship group, which also might help to mitigate against any effect of illusory biases. ADHD symptoms were associated with characteristics of the classroom friendship group (a peer-rated variable), though it did not act as a mediator of the association between ADHD and depressive symptoms in the current study.

Strengths include use of a representative school-based sample during the first year of secondary school with detailed information from multiple informants on different features of friendship, in addition to parent–child relationship quality. School life and transitions are important in adolescent mental health and may be particularly challenging for those with ADHD (Ford, 2020; Richardson et al., 2015).

Implications of this work include pinpointing quality of friendships and parent–child relationships as important to consider clinically in those with ADHD for reducing depression risk. Many peer relationship-focused interventions, which have mainly focused on peer acceptance and social skills thus far, have shown little success in children with neurodevelopmental disorders (Mikami, 2010). Promising directions for the development of enhanced programmes include those involving a parental component focused on dyadic friendship building (Gardner et al., 2019). Schools’ arrangements regarding awareness of friendship groups (e.g., keeping together or separating friends) are also important in ensuring children feel settled at the beginning of secondary school (Keay et al., 2015), and may need additional consideration in children with ADHD. Practical implications for children with ADHD in mitigating later risk of emotional difficulties may also involve focusing on interventions to strengthen parent–child relationships (Abikoff et al., 2015; Meinzer et al., 2020). Interventions aiming to improve parent–child interactions can have beneficial effects on the mental health of both child and parent (Sonuga-Barke et al., 2001).

## Conclusion

We found in a large school-based sample that ADHD symptoms were associated with subsequent depressive symptoms partly via decreased friendship quality, suggesting that this aspect of friendship is one potential mechanism by which ADHD symptoms increase risk for depressive symptoms. Positive features of parent–child relationships seemed to slightly alleviate the indirect effect via friendship quality, highlighting the importance of considering different sources of social support in the child’s life.

## Supplementary Information

Below is the link to the electronic supplementary material.

Supplementary file1 (DOCX 125 KB)
