# Supplementary material for: Investigating Friendship Difficulties in the Pathway from ADHD to Depressive Symptoms. Can Parent–Child Relationships Compensate?
Source: Res Child Adolesc Psychopathol. 2021 Mar 2;49(8):1031–41. doi: 10.1007/s10802-021-00798-w (PMC8222013; doi:10.1007/s10802-021-00798-w)
Supplement: Supplementary file 1 — Supplementary file1 (DOCX 125 KB) [file 10802_2021_798_MOESM1_ESM.docx]

**Supplementary Materials**

**Investigating friendship difficulties in the pathway from ADHD to depressive symptoms. Can parent-child relationships compensate?**

**Authors and Affiliations:** Victoria Powell^1^, Lucy Riglin^1^, Terry Ng-Knight^2^, Norah Frederickson^3^, Katherine Woolf^3^, Chris McManus^3^, Stephan Collishaw^1^, Katherine Shelton^1^, Anita Thapar^1^, Frances Rice^1^

 1.Cardiff University, UK

2.University of Surrey, UK

3.University College London, UK

**Corresponding Author:** Victoria Powell (UK Email: [powellv@cardiff.ac.uk](http://about:blank/))

Research on Child and Adolescent Psychopathology

**Supplement 1: Prediction of Missingness from Analysis Sample (Pre-Imputation)**

| **Predictor Variable (Baseline)** | **OR** | **95% CI** | **P-value** |
| --- | --- | --- | --- |
| Sex (1=male, 2=female) | 0.78 | 0.64, 0.95 | 0.014 |
| English as first language | 0.40 | 0.31, 0.51 | <0.001 |
| Black Minority Ethnic | 2.60 | 2.09, 3.24 | <0.001 |
| Free School Meals | 1.47 | 1.10, 1.96 | 0.009 |
| Special Education Needs | 1.61 | 1.23, 2.12 | 0.001 |
| Academic performance in English (Key Stage 2 test result) | 0.63 | 0.53, 0.76 | <0.001 |
| Academic performance in Maths (Key Stage 2 test result) | 0.70 | 0.61, 0.80 | <0.001 |
| Unauthorised school absences | 1.00 | 0.92, 1.08 | 0.976 |
| Authorised school absences | 1.03 | 1.00, 1.05 | 0.018 |

Within the children who participated at baseline (n=1712), logistic regressions between numerous predictor variables and being missing from the pre-imputation analysis sample (n=752) were conducted to establish predictors of missingness in this study. All the variables shown were included in the final missingness model for Multiple Imputation (Supplement 2). *OR* odds ratio, *CI* confidence interval

**Supplement 2: Multiple Imputation for Missing Data**

To address potential bias arising from missingness in our data, all analyses were conducted on an imputed dataset. Due to the variety of socio-demographic variables available in the STARS data which predict missingness (Supplement 1), we can assume that missing information is dependent on observed data. Therefore, we conducted Multiple Imputation by Chained Equations with the ‘ice’ command in STATA 13 to impute missing data for outcome and covariates (White et al. 2011). The imputation model included all analysis variables, recent contact with mother and father variables, the child’s social isolate status, and socio-demographic and school engagement variables that predicted missingness (Supplement 1), in addition to predictors of the exposure and outcome. This included earlier measures of child-rated depressive symptoms (taken in the first term of year 7) to predict the outcome of depressive symptoms (in the last term of year 7). The imputation model was used to predict missing data across 100 imputed datasets. Monte-Carlo errors were less than 10% of the standard error and FMI values were no larger than 0.71. After imputation, all analyses were run and estimates combined across the 100 imputations using Rubin’s rules.

**Supplement 3: Example of Classroom Friendship Group Questionnaire**

**Who hangs around together in your class?**

| **Are there some people in your class who hang around together a lot? Who are they? Write their names together on this piece of paper. Show as many groups as you can think of in your class.**  Some groups can have just 2 people. Some people might be in more than one group. You can use the list of people in your class to help you. Don’t forget to put your name on the map.  **Draw a circle around each group of people who hang around together a lot.**  Maybe some people don’t hang around in a group – you can put them in a circle on their own.  Write each person’s name clearly. If there are 2 people with the same first name put the first letter of their second name also.  To help you do this, the top box has an example of what this might look like once you are finished. This is a pretend example using made-up characters but you should do it for your class. |
| --- |
| 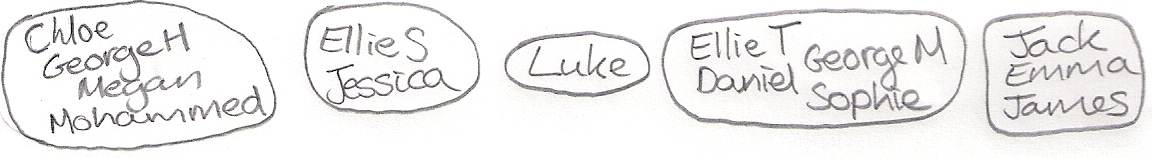 |
|  |

**Supplement 4: Example of the Guess Who Peer-Nomination Method**

**Guess Who?**

Read the following description of someone who **cooperates**. Look down the names of the people in your class. Tick the name of anyone you think fits the description. You can tick as many or as few people as you think there are. You can tick your own name if you think you cooperate. If you think nobody in your class fits that description, then you wouldn’t have any ticks.

**Cooperates** - *this person is really good to have as part of your group because they are agreeable and cooperate. They join in, share and give everyone a turn.*

| **NAME** | **CO-OPERATES** |
| --- | --- |
| Andi |  |
| Dylan |  |
| Ezri |  |
| Faith |  |
| Jennifer |  |
| Joseph |  |
| Kabeerat |  |
| Kamil |  |
| Keelan |  |
| Maria |  |

**Guess Who?**

Read the following description of someone who **disrupts**. Look down the names of the people in your class. Tick the name of anyone you think fits the description. You can tick as many or as few people as you think there are. You can tick your own name. If you think nobody in your class fits that description, then just go to the next description.

**Disrupts -** *this person has a way of upsetting everything when he or she gets in a group. They don’t share and try to get everyone to do things their way.*

| **NAME** | **DISRUPTS** |
| --- | --- |
| Andi |  |
| Dylan |  |
| Ezri |  |
| Faith |  |
| Jennifer |  |
| Joseph |  |
| Kabeerat |  |
| Kamil |  |
| Keelan |  |
| Maria |  |

**Supplement 5: Results for Subscales of the Friendship Qualities Scale (FQS)**

| **FQS subscale score** | **ADHD symptoms association with subscale (*b* (95% CI) *p*)** | **Subscale association with depressive symptoms**  **(*b* (95% CI) *p*)** | **Indirect effect via subscale between ADHD and depressive symptoms**  **(*b* (95% CI) *p*)** | **Percentage of total effect mediated** |
| --- | --- | --- | --- | --- |
| Companionship | -0.05 (-0.21, 0.12) 0.568 | -0.53 (-0.77, -0.28) <0.001 | 0.01 (-0.02, 0.04) 0.539 | 2.12% |
| Conflict | 0.17 (0.02, 0.33) 0.027 | 0.54 (0.30, 0.79) <0.001 | 0.04 (0.001, 0.07) 0.046 | 7.48% |
| Closeness | -0.17 (-0.31, -0.03) 0.014 | -0.24 (-0.51, 0.03) 0.086 | 0.02 (-0.01, 0.04) 0.197 | 3.59% |
| Help | -0.13 (-0.29, 0.02) 0.084 | -0.62 (-0.87, -0.37) <0.001 | 0.04 (-0.01, 0.08) 0.087 | 7.29% |
| Security | -0.23 (-0.40, -0.06) 0.008 | -0.54 (-0.79, -0.29) <0.001 | 0.05 (0.01, 0.08) 0.018 | 9.75% |

*N*=1712. *ADHD* attention deficit/hyperactivity disorder, *b* unstandardized beta, *CI* confidence interval

**Supplement 6: Moderation of Indirect Effects by Gender**

| **Mediator** | **Indirect effect (*b* (95% CI) *p*) in males** | **Indirect effect (*b* (95% CI) *p*) in females** | **Difference in indirect effect**  **(*b* (95% CI) *p*) in males versus females** |
| --- | --- | --- | --- |
| Quality: best friend | 0.03 (-0.01, 0.07) 0.147 | 0.08 (-0.003, 0.17) 0.059 | 0.05 (-0.04, 0.15) 0.254 |
| Quality: top three friends | 0.001 (-0.03, 0.03) 0.936 | 0.14 (0.02, 0.26) 0.020 | 0.14 (0.02, 0.26) 0.025 |

Indirect effects between ADHD and depressive symptoms via best friendship quality and top three friendships quality (tested simultaneously in multiple mediator model) in males, females, and the difference between genders (n=1712). *ADHD* attention deficit/hyperactivity disorder, *b* unstandardized beta, *CI* confidence interval

**Supplement 7: Moderation of Indirect Effects by Parental Warmth and Hostility**

| Moderator | Z-test of difference in indirect effect via **best friendship quality** at mean level of moderator versus mean+1SD (for warmth) or mean-1SD (for hostility) | Z-test of difference in indirect effect via **top 3 friendships quality** at mean level of moderator versus mean+1SD (for warmth) or mean-1SD (for hostility) |
| --- | --- | --- |
| Mother warmth | *z*=-0.02, *p*=0.981 | *z*=2.05, *p*=0.040 |
| Mother hostility | *z*=-0.35, *p*=0.725 | *z*=-0.07, *p*=0.945 |
| Father warmth | *z*=0.04, *p*=0.964 | *z*=0.28, *p*=0.783 |
| Father hostility | *z*=0.41, *p*=0.685 | *z*=1.73, *p*=0.084 |

Z-tests of difference between indirect effect via best friendship quality and top 3 friendships quality in the association of ADHD and depressive symptoms at mean level of moderator versus mean±1SD (n=1712). Parent variables were tested separately as moderators of a multiple mediator model (best friendship and top 3 friendships quality entered simultaneously). The model tested included interaction effects on the path between ADHD symptoms and friendship quality and on the path between friendship quality and depressive symptoms. *ADHD* attention deficit/hyperactivity disorder, *SD* standard deviation

**Supplement 8: Moderation by Mother Warmth of the Indirect Effect via Top Three Friendships Quality – Comparing Alternative Models**

| Model | Indirect effect (*b* (95% CI) *p*) at mean-1SD level of moderator | Indirect effect (*b* (95% CI) *p*) at mean level of moderator | Indirect effect (*b* (95% CI) *p*) at mean+1SD level of moderator | Z-test of difference in indirect effect at mean level of moderator versus mean+1SD |
| --- | --- | --- | --- | --- |
| Moderation of both paths of indirect effect | 0.06 (0.004, 0.12) 0.038 | 0.02 (-0.005, 0.05) 0.103 | 0.004 (-0.01, 0.02) 0.640 | *z*=2.05, *p*=0.040 |
| Moderation of ADHD to friendship path only | 0.04 (-0.0004, 0.09) 0.052 | 0.03 (-0.004, 0.06) 0.086 | 0.01 (-0.02, 0.05) 0.509 | *z*=1.27, *p*=0.203 |
| Moderation of friendship to depression path only | 0.06 (0.01, 0.11) 0.023 | 0.04 (0.0005, 0.07) 0.047 | 0.01 (-0.03, 0.06) 0.494 | *z*=1.49, *p*=0.136 |

Indirect effects between ADHD and depressive symptoms via top three friendships quality at mean level of moderator (mother warmth) ±1SD (n=1712). The models specified included a model with moderation effects on the path between ADHD symptoms and friendship quality and on the path between friendship quality and depressive symptoms (preferred model), a model with an interaction effect on the path between ADHD symptoms and friendship quality only, and a model with an interaction effect on the path between friendship quality and depressive symptoms only. *ADHD* attention deficit/hyperactivity disorder, *b* unstandardized beta, *CI*confidence interval, *SD*standard deviation

**Supplement 9: Results in Pre-Imputation Sample**

| **Friendship variable** | **ADHD symptoms association with variable (*b* (95% CI) *p*)** | **Variable association with depressive symptoms**  **(*b* (95% CI) *p*)** | **Indirect effect via variable between ADHD and depressive symptoms**  **(*b* (95% CI) *p*)** |
| --- | --- | --- | --- |
| Presence of friends | -0.05 (-0.08, -0.02) 0.004 | -0.21 (-0.60, 0.18) 0.291 | 0.02 (-0.03, 0.06) 0.470 |
| Stability: best friend | OR=0.95 (0.80, 1.14) 0.595 | -0.58 (-1.30, 0.14) 0.116 | 0.01 (-0.02, 0.03) 0.574 |
| Stability: top three friends | OR=0.99 (0.83, 1.17) 0.870 | -0.39 (-1.13, 0.35) 0.305 | 0.002 (-0.01, 0.02) 0.841 |
| Quality: best friend | -0.77 (-1.43, -0.12) 0.021 | -0.87 (-1.23, -0.50) <0.001 | 0.08 (0.004, 0.15) 0.040 |
| Quality: top three friends | -0.10 (-0.21, 0.01) 0.071 | -1.00 (-1.37, -0.62) <0.001 | 0.07 (-0.01, 0.14) 0.089 |
| Classroom friendship group: total difficulties | 0.21 (-0.15, 0.56) 0.248 | -0.07 (-0.31, 0.46) 0.707 | 0.003 (-0.03, 0.03) 0.855 |
| Classroom friendship group: cooperativeness | -0.02 (-0.03, -0.01) 0.001 | -0.26 (-0.65, 0.13) 0.196 | 0.03 (-0.04, 0.11) 0.396 |
| Classroom friendship group: disruptiveness | 0.02 (0.01, 0.03) <0.001 | 0.36 (-0.10, 0.83) 0.123 | 0.04 (-0.04, 0.13) 0.298 |

All results in pre-imputation sample (n=752) except for the association of ADHD and depressive symptoms (*b*=0.60 (95% CI 0.21, 1.00) *p*=0.002) are shown. ADHD and depressive symptoms remained associated when adjusting for baseline emotional problems (*b*=0.48 (95% CI 0.08, 0.87) *p*=0.018). ADHD and depressive symptoms remained associated when conducting non-parametric regression using the kernel smoothing method to account for any potential effect of skewness (*b*=0.62, 95% CI 0.13, 1.14, *p*=0.011). *ADHD* attention deficit/hyperactivity disorder, *b* unstandardized beta, *CI* confidence interval
